# How should robots exercise with people? Robot-mediated exergames win with music, social analogues, and gameplay clarity

**DOI:** 10.3389/frobt.2023.1155837

**Published:** 2024-01-12

**Authors:** Naomi T. Fitter, Mayumi Mohan, Rhian C. Preston, Michelle J. Johnson, Katherine J. Kuchenbecker

**Affiliations:** ^1^ Collaborative Robotics and Intelligent Systems (CoRIS) Institute, Oregon State University, Corvallis, OR, United States; ^2^ Haptic Intelligence Department, Max Planck Institute for Intelligent Systems, Stuttgart, Germany; ^3^ Rehabilitation Robotics Lab, Department of Physical Medicine and Rehabilitation, University of Pennsylvania, Philadelphia, PA, United States

**Keywords:** human-robot interaction (HRI), socially assistive robotics (SAR), physical HRI, exercise games, personal robots, rehabilitation robotics

## Abstract

**Introduction:** The modern worldwide trend toward sedentary behavior comes with significant health risks. An accompanying wave of health technologies has tried to encourage physical activity, but these approaches often yield limited use and retention. Due to their unique ability to serve as both a health-promoting technology and a social peer, we propose robots as a game-changing solution for encouraging physical activity.

**Methods:** This article analyzes the eight exergames we previously created for the Rethink Baxter Research Robot in terms of four key components that are grounded in the video-game literature: repetition, pattern matching, music, and social design. We use these four game facets to assess gameplay data from 40 adult users who each experienced the games in balanced random order.

**Results:** In agreement with prior research, our results show that relevant musical cultural references, recognizable social analogues, and gameplay clarity are good strategies for taking an otherwise highly repetitive physical activity and making it engaging and popular among users.

**Discussion:** Others who study socially assistive robots and rehabilitation robotics can benefit from this work by considering the presented design attributes to generate future hypotheses and by using our eight open-source games to pursue follow-up work on social-physical exercise with robots.

## 1 Introduction

The worldwide population is becoming less physically fit over time. For example, in the United States, 80% of adolescents and adults are insufficiently active (Piercy et al., 2018). This inactivity is accompanied by a reduction of metabolic health, an increase in cardiovascular morbidity and mortality, and an increase in the probability of diseases such as adolescent obesity, type 2 diabetes, and certain cancers (Must and Tybor, 2005; Young et al., 2016; de Rezende et al., 2014). On the other hand, physical activity as simple as standing up, walking, or taking exercise breaks comes with significant health benefits, such as lowering mean arterial blood pressure (Mainsbridge et al., 2014) and lowering insulin and glucose levels after meals (Dunstan et al., 2012; Bailey et al., 2016; Yates et al., 2020). Many technologies have previously been introduced to encourage people to be more active, from wearable devices (Kerner and Goodyear, 2017; Giddens et al., 2017) to phone or computer applications that support break-taking (op den Akker et al., 2014; Cooley and Pedersen, 2013); however, the prompts they deliver are often ignored, and even successful past studies of activity-promoting applications and devices show limited use and retention (Cooley and Pedersen, 2013). Compared to non-social or software-based encouragement approaches, social robots that are physically embodied are more likely to be able to encourage physical activity (Bainbridge et al., 2011); (Schneider and Kummert, 2018). These socially assistive robots have been evaluated as motivation companions for a variety of age groups, ranging from children (Swift-Spong et al., 2016; Guneysu and Arnrich, 2017; Akalin et al., 2019) to older adults (Fasola and Matarić, 2012; Avioz-Sarig et al., 2021).

**FIGURE 1:**
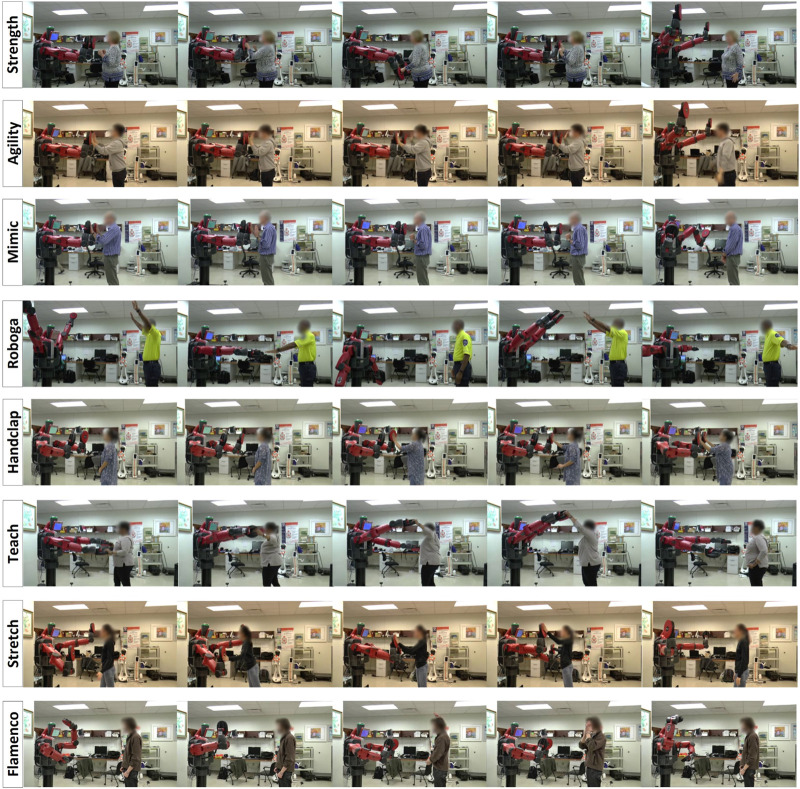
Illustrative frames of users playing the eight studied exercise games. Further details about each game appear in Section 3 and Fitter et al. (2020).

Related work has begun to consider ways to use robots to promote exercise, testing a range of approaches from activities involving wearable robotic exoskeletons (Ren et al., 2012; Riener et al., 2011) or physical interaction with robot end-effectors Rodgers et al. (2019) to social interactions with an encouraging robot (Fasola and Matarić, 2012; Kashi and Levy-Tzedek, 2018) or a musical robot (Baur et al., 2018). Findings to date show that adults of widely varying age all prefer exercise games (exergames) with a physically embodied robot over exercise with onscreen video of a robot (Eizicovits et al., 2018; Feingold-Polak et al., 2018). Past work in this space has typically employed either social interaction or physical interaction to support the needs of users, but seldom have robotic systems incorporated both. As pictured in Figure 1, the eight games analyzed in this article include six games that are socially and physically interactive in an intentional, dynamic, and high-energy way, thus following best practices for exergaming such as the dual flow model of mental and physical experience (Sinclair et al., 2009). Our past quantitative results demonstrated that these six social-physical games were generally more pleasant, enjoyable, engaging, cognitively challenging, and energy-inducing than the two studied games that lack physical interaction (Fitter et al., 2020). At the same time, not all of the physically interactive games were successful at inducing a positive exercise experience. This follow-up paper uses inductive analysis to dig further into the experience surrounding these exercise games and better understand why the most preferred games won user favor.

In the work presented here, our key research question is: *what attributes make robot-mediated exercise games successful at inducing a positive exercise experience?* We consider related literature on video games and assistive robotics in Section 2 to help identify potentially relevant game components. Section 3 describes our eight exercise games and their key components, in addition to the methods used to collect human experience information for each exergame. We move from the hypothesis-driven approach of our relevant past paper Fitter et al. (2020) to a more open-ended and hypothesis-generating inductive approach (centered on thematic analysis of qualitative data not considered in the past paper, with framing and context from the previously presented data) to better illuminate the path forward in human-robot exercise game interactions. The inductive analysis results in Section 4 include three main resultant themes: musical cultural touchstones, social experience, and gameplay clarity. We examine these results from the perspective of improving human-robot exercise gameplay for broader robotic systems and use cases promoting physical activity. Section 5 discusses what these results mean for human-robot exercise generally, with a particular emphasis on exploring cultural and social design elements and clear gameplay design. Key contributions of this work center on the identification of factors that can be leveraged in design and studied in future empirical studies to improve human-robot exergames.

## 2 Background

We draw essential context for the present work from related research on exercise games in rehabilitation robotics, design principles in video games as they relate to rehabilitation, and assistive robots.

Study of exergames involving robots and other technologies extends more than 30 years into the past. Early multimedia technologies for exergaming appeared in the video-gaming space, where activities from the 1987 *World Class Track Meet* for the Nintendo Entertainment System to the modern-day *Ring Fit Adventure* for the Nintendo Switch (and many games in between) have introduced ways for players to be physically active while gaming (Bogost, 2005; Nintendo, 2019). In addition to their presence in the consumer gaming space, exergames are a popular approach for encouraging general physical activity, promoting persistence, and providing entertainment in physical therapy. In the broad realm of physical activity, past work has focused on methods for evaluating behavior change (Abraham and Michie, 2008; Kok et al., 2016), which we could potentially use in future stages of this work. Other general physical activity research shows that planning routines together with users, providing instruction in physical activity, and reinforcing user effort are effective ways to increase human self-efficacy feelings toward exercise (Williams and French, 2011). In the rehabilitation space, particularly for arm exercises after stroke, researchers have proposed a multitude of exergames that involve peripherals ranging from handheld objects and joysticks (Goršič et al., 2017; Pereira et al., 2019) to robot arms and small mobile robots (Eizicovits et al., 2018; Rodgers et al., 2019; Guneysu Ozgur et al., 2018). Evidence to date shows that exergames that involve both social and physical interactions are likely to be most successful (Fasola and Matarić, 2012; Kashi and Levy-Tzedek, 2018). Accordingly, a fundamental idea in the design of our exercise games was the merging of these two types of interaction.

To design exergames that engage the user, we needed to consider and understand the extensive literature on games and gameplay. In gaming at large, a key challenge is designing activities that are both attractive and effective (Abeele et al., 2020). Vahlo and Hamari (2019) suggest that the five reasons why users choose to play video games are immersion, relatedness, fun, competence, and autonomy. For the topic of immersion in particular, Garone et al. (2020) emphasize the importance of touch and auditory stimuli. This observation aligns with our proposed use of social-physical contact and some of the game-design principles discussed in Section 3. Incorporating best-practice game-design ideas into exergames is crucial; these games need to be well-designed to overcome the monotony of the repetitive therapeutic exercises underlying most such activities. In exergame design, Luckner et al. (2018) further suggest incorporating components such as goal setting, avoiding competition, avoiding punishment, showing progress, and embedding exercises in daily routines to increase engagement. Active video games can promote different types of enjoyable energy expenditure (Lyons et al., 2011). In this past research, although energy expenditure was highest in fitness and dance games, enjoyment was highest in band simulation games that used licensed popular songs. This finding agrees with foundational research on conventional games that highlights the value of familiarity in making games appealing (DeKoven, 2013). Furthermore, the video-gaming literature highlights six forms of playfulness that roboticists could also seek to evoke: embodied investigation, constructive investigation, investigative storytelling, constructive storytelling, embodied storytelling, and embodied construction (Legaard, 2020). Another motivating factor in video games is the aspect of challenge, which can be broken into four components: physical, analytical, socioemotional, and insight (Vahlo and Karhulahti, 2020). These elements of playfulness and challenge informed our design and understanding of the investigated robot exergames. We return to key gaming ideas as framing in Section 3.2 as we introduce different exercise game components from our own work.

Our approach to encouraging exercise is also guided by past work that has leveraged social physically embodied robots as a unique way to motivate people. Socially assistive robotics Feil-Seifer and Matarić (2005) has shown potential for motivating and engaging people in a variety of tasks such as tutoring, physical therapy, and practicing the activities of daily living. One notable example used dance-based rehabilitation robotics for adults with Parkinson’s disease (Allen et al., 2017). This dance therapy with a robotic partner yielded improvements in gait, balance, and disease symptoms after 3 weeks of adapted tango classes. Exercise-based socially assistive robotics work showed that both patients and clinicians positively perceive robots used in conjunction with a treadmill for cardiac rehabilitation (Casas et al., 2019) and that a sociable robot to promote physical exercise is preferred over a more pragmatic and task-oriented system (Fasola and Matarić, 2012). Examples of socially assistive robots for exercise further include robots for walking practice promotion (Hamada et al., 2016; Mucchiani et al., 2017) and exercise activity teaching (Matsusaka et al., 2009; Moreno et al., 2016; Avelino et al., 2018). The humanoid robot NAO has been extensively used as a buddy that plays exergames with patients, such as imitation games for children (Pulido et al., 2019; Guneysu and Arnrich, 2017) and memory games for stroke rehabilitation (Sanchez et al., 2019). The Pepper robot has also been used an exercise buddy for post-stroke rehabilitation (Feingold-Polak et al., 2021) and as an autonomous empathetic exercise robot (Shao et al., 2019; Carros et al., 2020; Martinez-Martin and Cazorla, 2019; Cobo Hurtado et al., 2021; Akalin et al., 2019). In general, across user groups, imitation games appear to be the most common form of exergame performed by socially assistive robots (Pulido et al., 2019; Guneysu and Arnrich, 2017; Shao et al., 2019; Bäck et al., 2013; Görer et al., 2013; Matsusaka et al., 2009; Lewis et al., 2016). Our exergames with Baxter attempt to introduce a broader range of interaction types for robotic exercise-promotion systems. We also introduce new aspects to the interaction, such as a robot with a large workspace, dynamic robot motion capable of challenging a broader range of users, and direct social touch between the robot and the user.

## 3 Methods

### 3.1 Exercise games

As pictured in Figure 1 and described more thoroughly by Fitter et al. (2020), we developed eight exercise games that a user can play with a Rethink Robotics Baxter Research Robot [a human-sized upper-body humanoid robot with a face screen and built-in joint compliance (Rethink Robotics, 2012)]: the Strength, Agility, Mimic, Roboga, Handclap, Teach, Stretch, and Flamenco games. We were inspired by past work in robotic entertainment (e.g., Nuñez et al., 2014) to incorporate robot facial expressions, robot nonverbal behaviors, music, and audiovisual feedback into the games. The employed robot facial expressions come from Fitter and Kuchenbecker (2016)’s validated open-source Baxter face database, and the robot wears boxing pads on its end-effectors to facilitate safe contact. With feedback from experts in game design, rehabilitation robotics, physical therapy, and occupational therapy, these games were designed to promote moderate upper-limb activity and cognitive exercise. Brief descriptions of the games from Fitter et al. (2020) follow in descending order of user popularity, as reported later in this article:

• The Strength game is a boxing-training-like interaction during which Baxter holds up its end-effectors centrally and prompts the user to contact them.
• The Agility game challenges users to wake a “sleeping” Baxter by making rapid contact with its end-effectors.
• The Mimic game is a memory game during which the user teaches Baxter an increasingly long pattern of left-, right-, and both-handed claps.
• The Roboga game (an abbreviated spelling of “Robot Yoga”) requires the user to hold their arms aloft in poses demonstrated by Baxter.
• In the Handclap game, Baxter teaches the user a sequence of hand-clapping game motions by demonstrating the motions and then playing the game with the user.
• In the Teach game, the user can move Baxter’s arms to different positions to play and record musical chords mapped to its workspace.
• In the Stretch game, Baxter holds its end-effectors out wide, and the user must copy its pose and hit both of its end-effectors in each new pose.
• In the Flamenco game, Baxter teaches the user a sequence of dance moves to music, and the user then replicates the dance along with the same music clip.

To support additional understanding of the games, further explanation and demonstration of each activity is available in the Supplementary Video S1 included with this manuscript. We provide more discussion of key game elements matched to the video-game literature in the following subsection.

### 3.2 Exercise game components

From the perspective of common video-game (and exergame) mechanics, we had four components in mind while designing the games. These ideas are common in the related literature, although the perceived value of each component often does not match across the video-game and exergame contexts. Here, we explain how each component is connected to a subset of the games, and we return to these categorization ideas when discussing the inductive analysis later in this paper. The four identified components are: repetition, pattern matching, music, and social design.

#### 3.2.1 Repetition

Interestingly, though repetition is viewed negatively in the video-game literature (Desurvire and Wiberg, 2009), this element is essential in rehabilitation (Langhorne et al., 2011; Stevens and Tan, 2014; Bütefisch et al., 1995). This difference is one of the ways the investigated exercise games are different from games more generally; gameplay needs to be reevaluated as it moves from screen-based to embodied in the physical world, and as the goal shifts from pure entertainment to improving health. In accordance with the common rehabilitation need, four of the exercise games had an extremely repetitive premise (i.e., the robot struck a pose, and the human user matched the pose and possibly made contact with the robot’s end-effectors), as further described below. One of the main aspects that varied across these games was the workspace size; the order below reflects largest to smallest workspace used.

• Roboga: very large workspace; the robot struck four possible poses with fully extended arms, and the user matched the poses without contact
• Stretch: large workspace with a high number of possible robot poses (the result of mapping Baxter’s workspace into abstract musical quadrants), after each of which the user contacted the robot
• Strength: medium workspace with six possible robot poses (similar to boxing training), after each of which the user contacted the robot
• Agility: small workspace with just one central robot hand pose (the robot did not move at all, other than in passive response to the user’s hand contacts), which the user contacted repeatedly

The cycles of hand contact in these premises made them convenient for games designed with physical intervention in mind. The varying workspace could help to increase (or decrease) the level of spatial awareness required for the game, as needed for particular users or user populations.

Although the workspace and number of poses varied across these games, the mechanics of the portion of the game with active human input was very similar in premise, especially for the Stretch and Strength games. However, as discussed more in Section 4, user perceptions of these games varied widely.

#### 3.2.2 Pattern matching

Compared to the repetitive games above, these games built up to larger patterns of movement, as demonstrated by either the human user or the robot. This type of pattern matching is common in video games [e.g., in party games, minigames within popular franchises, and many games discussed in Lyons et al. (2011)] and rehabilitation (e.g., the socially assistive imitation games mentioned previously). The games in this category are listed in alphabetical order.

• Flamenco: robot lead, with no physical contact with the user. The robot demonstrated a sequence of three dance moves (out of four possible moves: a flourish or a clap to the left or right side) that the user then had to replicate. The pattern of dance moves grew by one move with each repetition and continued in this way until the game ended.
• Handclap: robot lead, beginning with a pattern of three handclaps (out of five possible moves: a clap across the body or on the same side with the left or right hand, or a two-handed contact) taught by the robot and then completed by the user and robot together twice. The game then lengthened by one handclap in each round, until the user made it to the fixed end of the game or lost the game by missing consecutive claps.
• Mimic: human lead and involving physical contact with the robot. The game began with a pattern of one move (out of three possible moves: left, right, or two-handed contacts) and built up a longer pattern until a mistake occurred.

The building patterns in these games, which the user needed to remember and replicate, linked them well to potential interventions that require cognitive effort, in addition to the physical requirements implicit in dancing or making contact with the robot.

#### 3.2.3 Music

Music games involved an element of music-making or a musical background theme as context. As with pattern matching, musical themes are common in both video games and rehabilitation. The work of Lyons et al. highlights popular musical games such as Rock Band and Dance Dance Revolution (Lyons et al., 2011), and we found several musical activities among the surveyed rehabilitation systems (e.g., Chen et al., 2017; Baur et al., 2018). The following list of these games appears in order from strongest/most ingrained to weakest musical premise.

• Teach: focused on music composition with the robot as an input device. The shoulder angle of each robot arm corresponded to one note in an abstract musical space, which the user could hear by turning the corresponding robot wrist. The user could lock in two-note chords to add to their composition by turning both of the robot’s wrists simultaneously. At the end of the song, the full composition played back along with the corresponding robot arm poses.
• Strength: this boxing-style interaction involved the theme music from the movie *Rocky* as a background soundtrack.
• Flamenco: this dance interaction occurred to the song “Malagueña.”
• Stretch: a musical chord played after each successful hand contact was registered, and playback at the end of the game shared the whole song while the robot held still, with playful incorrect notes for any poses that the user missed during the game.
• Mimic: three different drum beats played to correspond to (and register the success of) each demonstrated clap type (left hand, right hand, or both hands).

#### 3.2.4 Social design

Social interaction is a major element in both video games and rehabilitation. For example, in the Bartle taxonomy of video-game player types, “socializer” is one of the four major categorizations (Bartle, 1996), and socially assistive robotics is a full and burgeoning field in rehabilitation (Feil-Seifer and Matarić, 2005). Accordingly, seven of our eight games included interaction elements that were meant to make the activity social in some way. This type of design was central to all games aside from Teach, which was designed with the idea of the robot as a pure input device. The list of social games below appears in alphabetical order.

• Agility: the social analogue for this game was waking up a stubborn sleeping person. The robot snored and displayed a sleeping face at the start of the game. The snoring sound continued until the robot “woke up” (i.e., upon successful game completion). Baxter’s face changed from cool to warm colors in five discrete steps as the robot transitioned from a sleeping state to an awake state. The displayed robot face opened and closed the mouth to simulate snoring. As the participant made more contacts with the robot’s hands, the robot occasionally blinked, as if awakening briefly. If the participant paused for a sufficient duration, the robot went back to sleep. At the end of the game, if the participant succeeded, the robot stretched and yawned and the robot’s expression changed to one of joy.
• Flamenco: the social analogue for this game was dancing together with a dance partner. Baxter’s face color was yellow when it demonstrated the sequence and changed to purple when it waited for the participant, its partner, to replicate the sequence. The robot displayed a smiling expression on its screen throughout the interaction.
• Handclap: the social analogue for this game was playing childhood hand-clapping games with a friend, an activity which often transfers over to team-building or icebreaker activities. Baxter indicated that it was ready to clap hands with the participant by changing its face color from yellow to purple after demonstrating the sequence of hand-clapping motions. Baxter maintained a happy expression as long as the participant was physically interacting with its end-effectors. However, if the participant missed more than three moves, Baxter’s face displayed a sad emotion and the arms slumped in a dejected pose.
• Mimic: the social analogue for this game was playing pattern-building games with a peer, either with one another or with a toy like the pattern-matching game “Simon” (Wikipedia, 1978). Baxter changed its face color to green and nodded to indicate that it was waiting for the human user’s pattern. Baxter smiled and played a sound corresponding to each detected move during the participant’s teaching segments. Baxter’s face color changed to purple when it was replaying the learned moves together with the user. If Baxter lost a game, it performed a playful shrugging motion. In cases when the participant lost the game, Baxter raised its arms in joy and displayed a playfully impolite face with a tongue sticking out.
• Roboga: the social analogue for this game is doing yoga with an exercise partner. This game’s slow-paced stretching challenges the user to keep their limbs aloft, and Baxter’s presence functions similarly to a calm exercise partner. Baxter’s face changed from cool to hot colors to visualize the duration to hold each pose. The robot displayed a smiling expression on its screen throughout the interaction.
• Strength: the social analogue for this game was practicing boxing with a peer or coach. The robot moved its hands to a specific pose, changed the face color from blue to green, and played a bell sound to indicate it was ready for the participant to perform the boxing action. During the game, different levels of Baxter facial expression happiness (i.e., joyous, happy, neutral, sad) gave a running indicator of the user’s performance level, with the facial expression changing whenever a change in performance tier occurred. If the robot reached the sad face, the music also stopped, although the participant could still resume hand contacts to catch up and successfully finish the game. At the end of the game, the robot struck a pose whose level of celebration indicated the user’s game performance.
• Stretch: the social analogue for this game was a hand-tag or keep-away interaction during which a taller person (in this case, Baxter, which is tall relative to the average user) tries to playfully evade someone else and challenge them to reach far up and around. When a successful contact was detected, Baxter displayed a big smile and moved to the next pose. At the end during the song replay, Baxter displayed a smile with a purple face for all the successful contacts and a playful smirk with a red face for any failed contacts.

In addition to the above behaviors, Baxter randomly blinked its eyes during the entire study. When idle, the robot also randomly rotated its head left and right to create the illusion of observing its surroundings.

### 3.3 Exploratory study methods

We previously conducted an exploratory study to evaluate how users respond to exercise games with Baxter and how such games may fit into assistive applications. Eligible participants played a short segment of each game, immediately reported their perceptions of that game, and selected their favorite game to try again in a final longer free-play interaction. The University of Pennsylvania (Penn) IRB approved all study procedures under protocol 826370. The key research question guiding the inductive analysis approach presented in this paper asks how attributes that guided the exergame designs (i.e., repetition, pattern matching, music, and social design) and key themes observed during the study may connect to game success at inducing a positive exercise experience.

#### 3.3.1 Study factors

This experiment employed a within-subjects design that enabled all participants to experience all eight exercise games pictured in Figure 1. The experimenter read scripted instructions to each participant to prepare them for each semi-randomly ordered game interaction. When referring to each game, the experimenter used only a letter label (A-H), rather than the game name, to avoid unduly influencing participants’ interaction styles.

#### 3.3.2 Participants

We recruited participants using flyers in the Philadelphia area and emails to university listservs. Thirty-nine participants (20 male and 19 female) enrolled, gave informed consent, and successfully completed the study. One additional male participant completed only part of the study, as further explained in Fitter et al. (2020); we include data collected before his withdrawal and therefore have none of his demographic responses. During one participant’s session, we failed to record video of the first exercise game. Thus, for the video coding analyses later, the number of reported participants per game ranges from 38 to 40.

Participant ages ranged from 18 to 70 years old (aged 41.1 ± 18.7), where our notation represents the mean ± the standard deviation. 28 participants were affiliated with Penn. According to the demographic survey responses, the user group was made up of 21 individuals from science, technology, engineering, or mathematics (STEM) fields and 18 from non-STEM fields. On a scale from 0 to 100, participants ranked their experience with robots as 34.2 ± 30.0 and with Baxter as 21.2 ± 20.0. All participants possessed full function in their arms and hands and had normal or corrected-to-normal vision and hearing.

#### 3.3.3 Measurement

As detailed more thoroughly in Fitter et al. (2020), we recorded data about participant physical and cognitive state at the start of the study using the Box and Blocks manual dexterity assessment (BnB) (Mathiowetz et al., 1985), Beck’s Depression Inventory (BDI) (Beck, 1979), and (for older adults) the Montreal Cognitive Assessment (MoCA) (Nasreddine et al., 2005). We also recorded user height. Small adjustments were made to the gameplay based on each individual user’s arm span, motion speed capabilities (from the BnB performance), and cognitive function (from the MoCA score). Participant performance on these opening tasks was as follows: BnB scores of 60.1 ± 8.4 blocks moved per minute, BDI scores of 4.1 ± 5.3 where scores above 16 indicate depression (which could affect participant motivation), and older participant MoCA scores of 26.3 ± 2.6, where scores below 26 indicate mild cognitive impairment or dementia.

We also administered four further surveys during the study:

• An opening survey about pre-conceived notions of the Baxter robot based on a Unified Theory of Acceptance and Use of Technology (UTAUT)-centered questionnaire from Weiss et al. (2008) and additional questions from Heerink et al. (2009).
• A game evaluation survey after each game experience based on the Self-Assessment Manikin (Bradley and Lang, 1994), the NASA Task Load Index (TLX) (Hart and Staveland, 1988), enjoyment and engagement questions from past robotics work (Heerink et al., 2008), exercise level from the Borg perceived exertion scale (Heath, 1998), pain level from the Wong-Baker FACES pain rating scale (Garra et al., 2010), and a custom safety question from our own past work (Fitter and Kuchenbecker, 2018).
• A closing evaluation with the same robot evaluation questions plus general free-response questions.
• A demographic questionnaire.

Each of the self-rating questions was administered on paper using a continuous scale from 0 to 100. We verbally asked participants to select their favorite game after experiencing all exergames. We also videotaped the study and recorded data from Baxter’s sensors. The detailed results of the continuous-scale survey questions are not covered in this article since they were already analyzed in depth (Fitter et al., 2020).

#### 3.3.4 Study procedure

Each person came to the lab for a single 90-min session. Before the study interactions began, the participant completed the screening activities mentioned previously. The user received background information on Baxter and completed an opening survey. Next, the participant stood facing Baxter and played samples of the eight different exercise games in a semi-random order counterbalanced across participants. After each exergame, the user completed a survey about that game. The relayed instructions for each game and the general gameplay concept for each game are further explained in a video available as part of this article’s Supplementary Materials, and the source code for these exercise games is available at Fitter (2020). After the eight games, the user refreshed their memory of the game options by watching video snippets of all the games (in the same order as they had experienced the games), selected their favorite game, and entered a free-play mode during which they could play that game for up to 10 min. Lastly, participants completed a closing survey and a brief demographic survey. Participants received $20 for completing the study and up to $10 for transportation to and from the study site.

#### 3.3.5 Past analysis and results

As further reported in Fitter et al. (2020), our past work used analysis of variance tests on the questionnaire data to discover that games combining social and physical interaction were most pleasant, enjoyable, engaging, cognitively challenging, and high energy. Participant trust and confidence in the robot increased over the course of the study. There were also key differences in experience across age and gender; older adults experienced more exercise, energy, and engagement, and female users were more accepting of the robot than male users were.

#### 3.3.6 Current analysis

This article’s analysis focuses mainly on video coding, free-response data, and study video transcripts, as detailed below. As part of our early data analysis, we performed a *video-coding process* in which a trained annotator developed a codebook with the help of the research team. The videos were coded using MAXQDA 2018 (VERBI Software, 2018). The video annotation codes were designed to identify user verbalization and gestures that the robot could not automatically detect. The audio transcripts included three categories: comments made about the robot’s appearance or behavior, comments made about gameplay, and user verbalization to the robot. Items from the codebook for the videos and the audio transcripts appear in Supplementary Appendix SA and include general outcomes such as the number of times each participant attempted each game as well as game-specific events such as winning or making a particular mistake. All study videos were coded by the trained annotator, and a second trained rater evaluated a subset of the codes for which the primary annotator was unsure of their annotations. Any consistent coding differences were handled by discussion with the full research team.

To better understand the higher-level implications of these data streams taken together, we next used an *inductive approach to thematic analysis* (Maxwell, 2012; Creswell and Poth, 2016), following an iterative approach to discover patterns within the data and capture the qualitative richness of the phenomenon (Fereday and Muir-Cochrane, 2006). We performed iterative thematic labeling of all the text data (i.e., answers to free-response survey questions and transcripts of game-relevant participant speech during sessions) with labels related to the focus of each text snippet and the emotion of each text snippet. We then incorporated individual participant demographics, further codes from the video-coding process, and groupings of labels by game to discover patterns and cluster our labels into the themes presented in this work. A first research team member performed the initial thematic analysis, and a second team member reviewed the work and asked clarifying questions as needed until both parties agreed upon the proposed result. Any consistent questions or labeling differences were again discussed with the full research team. We also note favorite game selections of participants and self-reported exercise levels in the current manuscript as important context, but the main purpose of this follow-up work is to inform and generate future hypotheses.

## 4 Results

To build on our past findings and move toward a more holistic understanding of types of exergames, we present results from inductive analysis of both written and verbal user reactions to the exercise games. Parts of these analyses draw on the video coding to elucidate similar observations in a different way.

The breakdown of the participants’ favorite game helps to frame part of what is interesting about the results. The following list relays the frequency of each game’s selection as a participant’s favorite, in addition to a reminder of key game components discussed previously.

• Strength: 18 selections; repetitive game, musical design, social design
• Agility: 6 selections; repetitive game, social design
• Mimic: 5 selections; pattern-matching game, musical design, social design
• Roboga: 4 selections; repetitive game, social design
• Stretch: 2 selections; repetitive game, musical design, social design
• Handclap: 2 selections; pattern-matching game, social design
• Teach: 2 selections; musical design
• Flamenco: 0 selections; pattern-matching game, musical design, social design

Further, the games are intended to be exercise games, so it is important to consider their success in eliciting exercise alongside other factors. Accordingly, as previously reported in Fitter et al. (2020), the mean and standard deviation of the self-reported exercise levels (reported as *M* ± SD, with a maximum score of 100) for each game appear below, in order from most to least exercise elicited.

• Agility: exercise level of 54 ± 23
• Strength: exercise level of 39 ± 25
• Roboga: exercise level of 36 ± 23
• Handclap: exercise level of 27 ± 22
• Stretch: exercise level of 27 ± 21
• Flamenco: exercise level of 24 ± 21
• Teach: exercise level of 24 ± 22
• Mimic: exercise level of 21 ± 18

The participants in the study spanned wide ranges in age, cultural background, professional background, technical training, physical ability, and cognitive ability. Participant feedback reflected this heterogeneity; however, three common topics in particular emerged throughout the thematic analysis process, as detailed in the following subsections.

### 4.1 Musical cultural touchstones

The first of the identified themes, musical cultural touchstones, may be part of the story behind why games with similar repetitive premises could be perceived so differently. Based on the sorted qualitative feedback, the use of familiar music appears to be one main reason for the Strength game’s great popularity. Looking across musical games, the main category to which this theme applied, we see cases of implementation success, implementation failure, explicit missed opportunities, and implicit missed opportunities:

• Musical cultural touchstones done well: Strength
• Musical cultural touchstones done wrong: Flamenco
• Explicit missed opportunities: Stretch, Teach
• Implicit missed opportunities: Mimic

**FIGURE 2:**
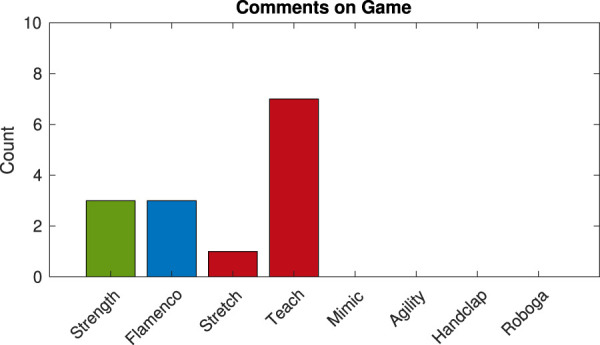
Number of verbal gameplay comments that occurred during each game summed across participants. The colors match identified music success groupings: green for musical cultural touchstones done well, blue for musical cultural touchstones done wrong, and red for explicit missed opportunities.

Games with musical touchstone comments tended to be the same as activities that led to spontaneous comments on the game generally, which we captured in the experiment video annotations. As shown in Figure 2, there were no verbal mid-activity game comments for any of the non-musical games (i.e., Agility, Handclap, and Roboga), musical games (specifically, Strength, Stretch, Teach, and Flamenco) collectively led to all 14 mid-play game-focused comments. Below we consider these notes alongside written free-response feedback provided by participants, with an emphasis on comments that focused on the musical element.

#### 4.1.1 Game comments for musical touchstones done well

The *Strength* game, the standout example of a musical cultural touchstone done well, garnered 17 comments overall (a mix of spoken and written) focused on either enjoying or recognizing the music from *Rocky*. This movie is famous for its training montage set to the song “Gonna Fly Now” and picturing famous landmarks in Philadelphia, where the study took place. Many participants labeled the *Rocky* reference explicitly [e.g., “Hitting the robot while listening to Rocky Music” (Subject 32), “The Rocky punching one was the best!” (Subject 9)]. Video transcripts likewise included references to the movie while speaking to Baxter, e.g., “This is…are you trying to train me like the guy (Rocky)?” (Subject 2). In the end, the Strength game was the most popular of all the games, with 18 participants selecting it as their favorite. It also led to significantly more exercise than any other game aside from Agility, as further reported in our past work (Fitter et al., 2020). Verbal comments about the game doubled down on this success at energizing participants, e.g., “Hahaha! This is fun!” (Subject 9), “That’s it?! Man!! I could go on for days!” (Subject 9), and “I like that one. It (…) pumped me up!” (Subject 2).

#### 4.1.2 Game comments for musical touchstones done wrong

Compared to the above case, using a musical cultural touchstone that is not recognized by the user base misses the mark. The *Flamenco* game was based on a well-known guitar song, Malagueña, but the participants seemed to be unfamiliar with it. Only three people commented on the song, using the quick descriptors “great music” (Subject 37), “good music choice” (Subject 4), and “cheesy” (Subject 39). Relative to this count, more game comments (five in total) focused on participants’ self-assessed weaknesses at dancing. In the end, although it did not lead to any notes on technical deficiencies, Flamenco was not chosen as a favorite game by any participants. It also was among the bottom group of games for exercise induction. Spoken comments on this activity varied from “That was hard!” (Subject 27) to “That’s it? This is easy! (laughter)” (Subject 2), or even prescription to someone else as a better potential user [e.g., “my daughter would love that” (Subject 34)].

#### 4.1.3 Comments for games with missed musical touchstone opportunities

Among other musical games, nine comments indicated a desire for more familiar music. Responses to the *Stretch* game included “I want something I recognize. Even if it is a children’s song” (Subject 7), “It didn’t sound like anything though…” (Subject 20; the one verbal response), and “Music was not very celebratory” (Subject 39). One participant noted that the *Teach* game “may be more exciting if (it) had a popular tune to compose or song to follow” (Subject 6). Experiences were not fully one-sided; some verbal feedback on the game was positive [e.g., “Kinda cool to play with!” (Subject 10)] while other notes showed displeasure [e.g., “I feel like this is really weird” (Subject 21), “that’s not pretty” (Subject 9)]. In the end, two participants chose each of the above games (Stretch and Teach) as their favorite, and both games were among the lowest tier of games for exercise promotion.

No explicit comments addressed the *Mimic* game music; since it entailed only drum sounds, it may be perceived as belonging to a different musical realm than the other musical games. Regardless, game comments for the Mimic game uniquely hinted at feelings of accomplishment or self-efficacy; some participants liked the game because “you kind of taught (the robot) something (during the Mimic game)” (Subject 33) or rose to the challenge, proclaiming “I beat him! After the fourth time” (Subject 27). At the same time, Mimic was the third most popular game (with five selections), although it appeared to be more cognitive than physical in terms of exercise; it tended to be the lowest in terms of exercise level production. Incorporation of a strong musical touchstone might make this exergame even more effective.

### 4.2 Social experience and immersion

The second overall theme seems to be another potential reason for the disparate perception of games with similar mechanics. This topic, social experience and immersion, is a shorthand for how clearly the user understood and accepted the intended social role of Baxter (the social analogue) in each game, as described in more depth in Section 3.2.4. Immersion is a common video-game idea that aims to describe a loss of self-awareness and richness of interaction experience (Desurvire and Wiberg, 2009; Jerzak and Rebelo, 2014). For exergames, past work supports the idea that flow is likewise a helpful feature (Huang et al., 2018); for example, becoming engrossed in an activity could help users forget that they are carrying out repetitive motions. Looking across social games, participant comments hinted at three different tiers of implementation success:

• Social analogue understood and accepted: Agility, Roboga, Strength
• Social experience understood, but not accepted: Flamenco, Handclap
• Social experience not clearly understood: Mimic, Stretch

**FIGURE 3:**
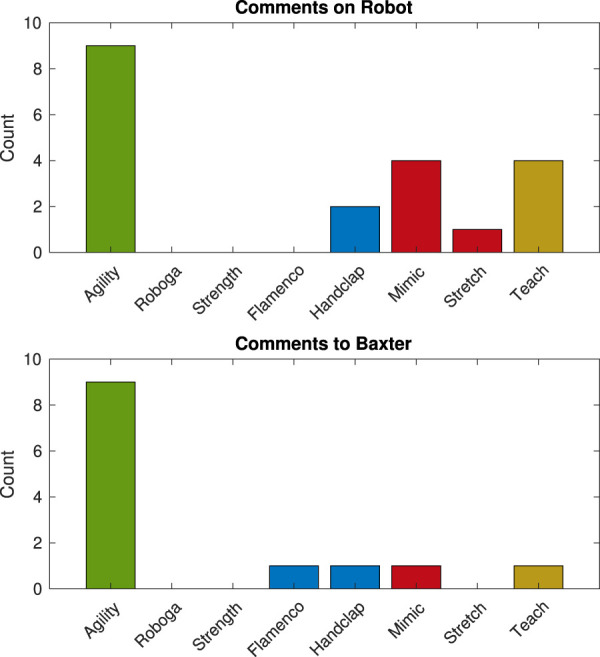
Number of verbal comments about and to Baxter made by all participants during each game. The colors match social engagement success groupings: green for social analogue understood and accepted, blue for social analogue understood but not accepted, red for social experience not clearly understood, and yellow for no social analogue.

For some games, players recognized the analogy but failed to buy into the interaction paradigm, and for other activities the underlying social idea was lost altogether. On the other hand, sometimes both of these aspects of the social interaction design went smoothly. Below, we share evidence that led to the proposed sorting of games alongside incidence levels of comments about and to the robot, as shown in Figure 3, which seem to have inherent connections with the general social experience. While considering social interactions with the robot, it is worth briefly noting that although the study script used “it” pronouns to refer to Baxter, most participants used male pronouns to describe the robot; similar tendencies to gender robots have also appeared in past related work (e.g., Stroessner and Benitez, 2019; Søraa, 2017).

#### 4.2.1 Social experience and immersion success

The thematic analysis indicated that Agility, Roboga, and Strength were all successfully interpreted as their intended social analogues and seemed to immerse users effectively. During the *Agility* game, two users explicitly labeled the interaction as similar to waking up a deeply slumbering family member. One compared the interaction to “waking up my son, who loves to sleep (and is) a bit difficult to wake up” (Subject 27), and another noted “He’s like one of your child…your children. Get up! Get up! You can’t sleep!” (Subject 16). Another participant mentioned that the “robot is a heavy sleeper” (Subject 20). Similar to typical behaviors while trying to wake a friend or family member from sleep, this game led to the most instances of mid-game talking to the robot (nine of 13 total instances across games) and about the robot (nine of 20 instances). Much of the dialog with the robot was playful, such as “I should’ve just thrown some water on him. That’d be easier” (Subject 30) and “Wake up! Don’t make (me) use bad language!” (Subject 31). The smooth social experience of the game may have contributed to the popularity of the activity (second most favorite of the games, with six selections) as well as the high exercise level; Agility led to significantly more exercise than any other game, as further reported in Fitter et al. (2020).

*Roboga* likewise led to interpretation of the activity as intended, as well as to creation of an immersive experience. Several participants labeled the activity as yoga explicitly [e.g., “Reminded me of yoga” (Subject 18), “It’s like yoga” (Subject 10), “It was very calming. It felt like yoga” (Subject 15)]. Further, one user expressed interest in the broader use of the robot as a yoga partner during this game [i.e., “I think it could be fun to do yoga w/Baxter” (Subject 24)], and another person declared “Thank you, Baxter!” (Subject 27) at the end of the activity, in a similar way that one might do at the end of an exercise class. Unlike the Agility game, Roboga did not lead to any instances of the participant talking to or about Baxter during the game; however, this is authentic for the intended type of exercise experience. It would be unusual for someone to begin chatting with a classmate or instructor during yoga class. Roboga led to four selections as favorite game and a moderate amount of exercise (i.e., more than Mimic, and not significantly less than Strength).

Finally, the *Strength* game seemed to succeed in terms of both social experience and immersion. Participants noted the boxing premise specifically in comments like “I want Baxter to be my boxing coach” (Subject 24), “Boxing with Baxter was engaging” (Subject 27), and “That was fun! They should call it [the robot] “Boxter” (Subject 5). As might be expected with the boxing context, participants appeared to be more focused on the physical aspect of the game than bantering with the robot during the activity; there were no mid-activity comments to or about the robot. This flowing social experience may have bolstered the popularity of the game; as mentioned previously, Strength was the most common choice of favorite game, and it led to one of the highest exercise levels.

#### 4.2.2 Social experience without immersion

Two games, Flamenco and Handclap, fell into this next category of activity recognizability without full buy-in. For *Flamenco*, people understood that they and the robot were dance partners, noting “I have to brush up on my dance moves!” (Subject 40) and “I performed just like in real life - never could follow someone on the dance floor”: (Subject 38). But something felt off about the interaction, e.g., one user commented “It feels a bit awkward dancing so slowly” (Subject 7); technical limitations prevented implementation of faster robot dancing. Perhaps in part because of this lack of immersion, Flamenco led to just one comment to the robot during gameplay (in addition to being the least favorite and among the lowest inducers of exercise).

The *Handclap* game was easy to interpret as such, but something likewise felt wrong in its flow. One participant noted that it is “easy to play (a) clapping game with (a) human, but (they) had difficulty remembering Baxter’s routine or predicting which hand (would go) which direction” (Subject 2). Relatedly, one user needed to think so hard about the instructions that they repeated key ideas back to themselves aloud [e.g., “When the hands are up top he’s done. Okay” (Subject 27)], and another participant misunderstood Baxter’s motions, asking, “Is it a hug? can I get a hug?” (Subject 31). Dialog to and about the robot sometimes occurred during this game, but it usually centered on the user’s lament about or surprise at their own bad performance, e.g., “Baxter dude, I didn’t do too good” (Subject 38) and “Oh! Baxter! Why do you look sad!?” (Subject 31). Two participants chose Handclap as their favorite game, and this activity was among the lowest tier of games for exercise promotion, although it tended to be near the top of this group.

#### 4.2.3 Social experience not well understood

In other games, namely, Mimic and Stretch, the analogy to everyday social experiences was not clear. Participants still found enjoyment in *Mimic*; for example, one user mused that they “enjoyed being able to teach Baxter” (Subject 24). This observation indicates some understanding of the activity premise, but not a strong connection to the light-up Simon toy analogue. At the same time, this game inspired a moderate amount of commenting about the robot’s end-of-game response, which playfully teased participants in different ways. Nevertheless, Mimic was one of the most common game favorites, although it did not lead to much physical exercise.

The *Stretch* game failed to conjure any successful ties to hand-tag-like childhood games, although one participant saw the potential for a clearer analogy, noting that “avoiding my high-five would have been more engaging” (Subject 8). There was only one comment about the robot during the gameplay [i.e., “Oh! It didn’t like that” (Subject 1), when a hand contact failed to register correctly]. Interestingly, this is one of the few times when a user labeled Baxter with the pronoun “it.” As highlighted before, Stretch was near the bottom of the game group for both selections as favorite and exercise production. A stronger social analogue could increase the effectiveness of both of these games.

### 4.3 Gameplay clarity

A final influential theme in the thematic analysis, and apparently a key to user experience, was feedback on gameplay clarity. It seemed that certain aspects of gameplay clarity could make or break the success of activities in terms of user experience and induced exercise. A subset of games appeared to be highly clear in terms of gameplay, others had selected flaws that did not prove to be dealbreakers, and the final group were so flawed that issues in gameplay clarity interfered with us learning much else about the game. Participant comments indicated the following breakdown:

• Gameplay clarity done well: Flamenco, Roboga, Stretch
• Explicit gameplay clarity improvements noted: Agility, Mimic, Strength
• Gameplay clarity done badly: Handclap, Teach

This split may indicate that the gameplay mechanics do not need to be perfect (in fact, the three most common favorite game selections are in the middle category), but there is a level of confusion and difficulty from which there is no redemption (i.e., the final category).

**FIGURE 4:**
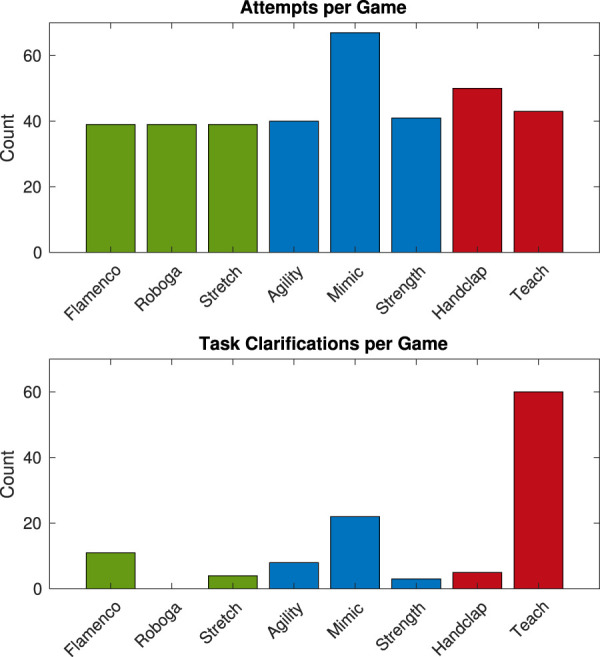
Number of attempts and task clarifications for each game. Note that the expected number of attempts for a participant (if all goes correctly) is 1, which would lead to 39 or 40 total attempts for each game. The colors match identified gameplay clarity groupings: green for gameplay clarity done well, blue for explicit gameplay clarity improvements noted, and red for gameplay clarity done badly.

We consider these clarity groupings alongside the number of times participants needed to try the game to achieve a successful interaction and video coding results that showed objectively how many times each participant asked clarifying questions about the task, as shown in Figure 4. These data streams are supplemented by counts of the different types of errors participants made while playing each game, as reported alongside results below. These pieces of information taken together reveal more about what types of confusions are forgivable and how our exercise games might be improved in the future.

#### 4.3.1 High-clarity game success rates and errors

Based on the inductive analysis, the Flamenco, Roboga, and Stretch games all seemed to be high-clarity games. For each one, only a few corresponding critiques related to clarity issues, which we take to mean that the mode of play for each game was (at the very least) not unclear to participants. Most of the *Flamenco* comments centered on potential improvements to the feedback provided by the robotic system [e.g., “It was hard to tell with the music when to start the dance motions” (Subject 9)]. For *Roboga*, one comment showed potential for a clearer match between the user’s body and Baxter’s pose, i.e., “I think the red pads were like the palm of the hand to help determine hand position” (Subject 37). The *Stretch* notes included a suggestion to increase the system volume slightly.

##### 4.3.1.1 Task clarifications

The above observations from the raw data align well with the count of task clarification codes that were noted in the video recordings for each activity. Roboga had the fewest task clarifications, with zero. Stretch was among the three lowest with four of this code, and Flamenco was closer to the middle of the group with 11. These clarification requests did not directly reflect problems in gameplay clarity, but there is likely a close connection between observed clarity issues and participant questions to check that they are playing the game correctly; the latter reflects the meaning of this code. No participants needed an additional attempt for any of this grouping of games.

##### 4.3.1.2 Success rates and user errors

As far as game completion success goes, every participant reached the end of the Flamenco game, but we observed assorted dance motion errors performed by participants. In ten cases, users added an extra move while dancing with the robot (across participants, 0.3 ± 0.8 extra moves, with a maximum of four for any given participant). Participants also missed a dance move 29 times (over participants, 0.7 ± 1.8, with a maximum of eight) and performed an incorrect move in their dance sequence 44 times (over participants, 1.1 ± 2.2, with a maximum of ten). As shown by these error distributions, Flamenco mistakes arose more frequently for just a subset of the participants even though the game premise was clear. Added feedback about the correctness of dance moves might have reduced the occurrences of these errors.

All participants successfully completed the Roboga game, but we noticed two types of errors wherein participants either did not follow the verbal game instructions or did not follow Baxter’s example. In 34 instances overall, participants failed to relax their arms during the brief relaxation period between sets of held poses. Across participants, this constituted 0.9 ± 1.6 failures to relax, where three was the maximum number for any one participant. This behavior actually just led to extra exercise, but it could be considered one type of participant error, likely stemming from the fact that Baxter’s arms cannot hang straight down as human arms can. There were also 52 cases when the participant’s pose did not accurately match Baxter’s pose, almost always due to differing orientations of the palm (across participants, 1.3 ± 1.2 mistakes, with a maximum of four for a single participant); this type of error matches the participant comment about hand orientation ambiguity.

In the Stretch game, participants were uniformly successful at playing and completing the game. The only recorded error during this game was an error in the robot’s sensing strategy; during one move, the participant seemed to contact the robot in the correct way, but Baxter’s accelerometer-based contact detection strategy did not successfully identify the hand contact. This game appeared to be easy to play, but not necessarily engaging.

#### 4.3.2 Medium-clarity game success rates and errors

In the next category, Agility, Mimic, and Strength seemed to be medium-clarity games. For the *Agility* game, comments focused on being unsure how hard or fast to hit the pads, as well as wanting a more continuous spectrum in the facial responses to see their progress, e.g., “The lack of a continuous color scale makes it difficult to see if progress is being made” (Subject 8). Most of the *Mimic* comments were about clearer cues for who had lost or won the game, and when this occurred. The *Strength* game comments focused on the other sounds making it hard to hear the bell cue, and thus, their reliance on the facial cues for timing, such as “Could not hear bell to strike pads. Watched for green face” (Subject 23).

##### 4.3.2.1 Task clarifications

The task clarification rate matches the above list closely with one exception. Agility and Strength both led to very low numbers of task clarification instances (eight and three, respectively). On the other hand, Mimic led to the second highest number, at 22. This result may indicate that although the idea behind the Mimic game (pattern teaching, matching, and building) was familiar, the underlying mechanics were not clear enough (i.e., 12 users had clarifying questions about this game, with up to four questions coming from the same person). Similarly, 18 participants needed a second attempt when playing the Mimic game, after misunderstanding the low-level game mechanics during the first try. No users needed more than one attempt to play the Agility game, and one user needed a second try at the Strength game.

##### 4.3.2.2 Success rates and user errors

In terms of game completion success, in the Agility game, only one of our 40 participants failed in the central task of waking the robot up. This individual tried the activity twice and then decided to give up at the game, resulting in a 2.5% failure rate overall. Seven participants exhibited one additional behavior that revealed potential confusion about the gameplay; they continued to hit Baxter’s arms even as the robot began yawning, stretching, and waking up. This error sometimes seemed to be playful and deliberate.

30 study participants won their final trial of the Mimic game, and nine lost. Of these recorded losses, six were due to the participant forgetting their own pattern and making a mistake, and three were due to Baxter declaring victory after a false positive or false negative hand contact. An additional user made a clapping pattern error simultaneously with a robot error, leading this trial to be recorded as a win for the human user. Thus, we consider seven of the trials true user losses; this 17.9% failure rate was the highest of any game. At the same time, this game was the third most popular overall; we discuss why this might be the case later in the paper.

Baxter’s end-of-game facial expression and body language was a high-level indicator of participant score in the Strength game. 39 users achieved the highest score bracket possible, and one participant received the “neutral” final reaction from Baxter, indicating that they just barely completed the game successfully. This game also led to one unexpected participant tendency: several users struck Baxter in a rhythmic pattern matching the beat of the *Rocky* song, rather than delivering a one-two punch when cued by Baxter. This behavior was similar enough to the expected gameplay that this discrepancy did not lead to difficulties completing the game.

#### 4.3.3 Low-clarity game success rates and errors

The final low-clarity category included Handclap and Teach. Based on the inductive analysis, there seemed to be multiple layers of trepidation about required input from the user and correct performance of these games. The comments for the *Handclap* game focused on the cross-clap being difficult to differentiate from the normal single clap and the transition from demonstration to collaborative clapping being unclear. For the Handclap game in particular, this lack of clarity overran the familiarity of the game premise. Participants had particular difficulty distinguishing between types of claps [e.g., “I kept confusing the cross hit for the regular one” (Subject 33)]. The Teach game comments focused on general uncertainty about musical performance skill, not being able to hear the note before selecting it, and not being able to listen to what they have so far for the song. *Teach* led to different points of confusion and frustration, from “(I) was a little confused this time regarding how to make the notes continue” (Subject 19) to “There was no way to hear a chord w/o recording it. Frustrating” (Subject 13) (a point of critique for three different users).

##### 4.3.3.1 Task clarifications

In this game grouping, we see the incidence of Teach game clarifying questions matching well with the thematic analysis. Teach led to the highest overall number of clarifying questions, with 60 total. On the other hand, Handclap did not lead to many (just five total). This observation may show that while the game itself is not hard to play, there is an inherent flaw in the way it was implemented, as also hinted at in the quotes above. On the other hand, the Handclap game did require an outsized number of tries compared to all other games; 12 participants required a second chance while trying this game, whereas only three participants needed an extra try for Teach.

##### 4.3.3.2 Success rates and user errors

In the Handclap game, we noticed that some participants lost, some claps were missed, and participants did not always contact the robot with the correct hand. 35 participants successfully reached the end of the game, and three lost the game to Baxter. This game’s 7.9% loss rate was the second highest of all the games. Even for participants who won the game, some claps were missed; users did not lose the game unless several consecutive claps were missed. 34 total claps were missed by participants collectively. Across participants, this constituted 0.9 ± 1.4 claps missed, where seven was the maximum number of claps missed by a single participant. A more common error was contacting Baxter with the incorrect hand. Overall, 140 claps during the collective trials were performed with the incorrect hand (across participants, 3.7 ± 5.3 incorrect claps, with a maximum of nineteen wrong claps). These error distributions show that mistakes arose commonly across participants during the Handclap game.

The Teach game was very collaborative in nature and had no concrete performance objectives, so we evaluated how many chords were recorded and explored by participants (a measure of how much participants engaged with the game). Song length was 17.4 ± 19.4, where songs ranged from three to 121 recorded notes. Most participants tried deliberately to create a song, while a few explored chaotically.

## 5 Discussion

In conjunction with framing information on favorite game selections, induced exercise levels, and video annotations, the inductive analysis results paint a picture of why certain games were more successful than others in terms of user experience and induced exercise levels; they also suggest promising ways to design robot-mediated exercise activities in the future. In this section, we discuss key findings from the inductive analysis results and highlight potential takeaways (typeset in italics) for the design of future human-robot exercise games. For those interested in our exercise games in particular, we also encourage a close review of the game improvement suggestions in Supplementary Appendix SB. We conclude with key strengths and weaknesses of the work, followed by final thoughts.

### 5.1 Musical cultural touchstones

The music-focused theme that arose from the inductive analysis is a possible explanation for why the Strength game was so successful on both the user-experience and the exercise-level fronts, despite its simple and repetitive premise. The great popularity of this game goes against the idea of repetitive games leading to disengagement, which implies the possibility that building cultural references into robot-mediated exergames will improve user engagement. This observation is similar to the finding of Lyons et al. (2011) that games using licensed popular songs were most enjoyable to users. It also aligns well with past work on music in exercise generally, which shows that music can help to heighten physical activity levels (Clark et al., 2016; Karageorghis and Priest, 2012; Wiemeyer, 2012), and that the selected music should be congruent with characteristics of the user, task at hand, and workout goals (Karageorghis, 2020).

Other games did not succeed in their musical references (i.e., Flamenco) or missed opportunities for introducing cultural references (i.e., Mimic, Stretch, Teach). The song Malagueña was not nostalgic for most participants, although it might be relevant in other cultural contexts; for example, one employee who worked across the hall from the study site noted that they loved hearing the song playing due to their Cuban cultural heritage. Disappointed comments on the recognizabilty of Stretch and Teach game music demonstrates an important focus on cultural touchstones for game success. Taken together, these results support the idea that musical cultural references can be used as a method for rapidly building game enthusiasm and engagement. In the cases of our exercise games, we hypothesize that games using more familiar music for the cultural context of the study (in our case, a large urban center in the United States) would inherently be more popular than those without this musical connection. This idea could be tested further in follow-up work, both within and beyond our use case.

### 5.2 Social engagement and immersion

Data surrounding this second theme may reveal why the Agility game was the second-most-popular activity, despite being even simpler than the most-popular Strength game. Again, this game was extremely repetitive and had one of the simplest premises in our set of games (waking up the robot). We believe the key for Agility may be found in the relatedness and immersion themes from video-game research (Vahlo and Hamari, 2019), which seem to tie to the social context recognition and flow experience observed in Agility (as well as the previously discussed and popular Strength game and the reasonably popular Roboga game).

The social analogue underlying the Agility game was quite clear to participants, and several users directly articulated the interaction metaphor underlying the activity. The same was also true for Roboga and Strength. One difference across these socially engaging games was the amount of instigated speech to and about Baxter as a result of gameplay; Agility commonly prompted these actions, while Roboga and Strength did not. At the same time, these user behaviors match typical norms in the corresponding social scenario analogues.

Flamenco and Handclap were recognizable as their design metaphors, but something was off for each of them, which seemed to prevent a fully immersive experience. In Flamenco, this missing link may have been a result of the song selection; for example, regionally relevant line dances like the Cha-Cha Slide or the Wobble may have been better choices for promoting a flow experience. Users recognized the similarity of Handclap to childhood hand-clapping games, but without the self-clapping motion common to these games and the typical back-and-forth flow (design choices made to smooth the sensing system side of the game and make the experience sufficiently different from other games in the set), something seemed to be amiss.

The final socially designed games were not perceived in a nostalgic social way at all. Participants understood that they were teaching a pattern or following Baxter’s actions for Mimic and Stretch, respectively, but the activities did not conjure up any connection to familiar experiences. One way to inspire a more social connection to the Mimic game could be to lean into the “Simon” game analogue (for example, by having four action options, using the colors red, blue, green, and yellow to each correspond to one move, and including “beep” sounds akin to those of the original electronic game). As suggested by participants, a more playful behavior paradigm such as Baxter overtly trying to avoid the user’s hand may have made the Stretch game more successful.

A final social engagement note is that our results show that *users will interact with a robot in a social way even if it is nonverbal.* In socially assistive robotics, robots most commonly use natural language to communicate. The addition of speech to a robotic system can be a double-edged sword; it can add clarity to communication, but it also tends to increase the user’s expectations of what a robot is capable of (often beyond the realm of the realistic) (Kwon et al., 2016). Even without speech, participants reacted socially to Baxter (particularly in the more challenging games) with chiding, encouragement, pleasantries, apologies, banter, and gendering. We believe part of this success was due to the system’s facial expressions (easily customized with the face screen), as well as other social cues integrated into the games. This finding echoes existing knowledge from human-robot interaction; past studies (e.g., Fong et al., 2003; Epley et al., 2007; Krach et al., 2008) likewise indicate that humans tend to react in a social way to robots whether or not these systems are intentionally designed to be social. A new aspect of the current work is gaining this type of understanding for social exercise gameplay with industrial collaborative robotic systems like the Baxter robot in particular.

### 5.3 Gameplay clarity

The final theme from the inductive analysis showed that Flamenco, Roboga, and Stretch were clear games in terms of the users naturally understanding what they need to do for gameplay. Agility, Mimic, and Strength were partly clear with moderate potential for improvement, and Handclap and Teach were not clear. In general, high-clarity games tended to be very similar to the traditional imitation-based interactions that are commonly used in socially assistive robots (Pulido et al., 2019; Guneysu and Arnrich, 2017; Shao et al., 2019; Bäck et al., 2013; Görer et al., 2013; Matsusaka et al., 2009; Lewis et al., 2016). At the same time, most of the top-selected favorite games were in the middle clarity category (i.e., all but Roboga). This contradiction may signal the importance of having compelling references and social experiences as part of gameplay design, in addition to hinting that minor clarity problems can be forgiven within the context of a game that has other appealing design elements.

Our results show that participants were generally successful at completing the exercise games. The majority of participants completed or won every exercise game. Across all participants, we recorded only thirteen instances of participants losing games. Additional robot errors (seven robot sensing errors in the Mimic game and one in the Stretch Game) and participant errors (continuing to hit the robot after the Agility game had ended and making movement errors in the Flamenco, Handclap, and Roboga games) highlight specific opportunities to update our robotic system and improve the guidance that participants receive during games. For example, feedback on the correctness of Flamenco dance moves and Roboga poses would likely lead to higher movement accuracy; a camera-based markerless motion-capture system could be used to enable real-time assessment of such movements (Mohan et al., 2021).

### 5.4 A note about the mimic game

Among the four most popular exercise games (i.e., Strength, Agility, Mimic, and Roboga), the preference of users for Strength, Agility, and Roboga is well explained by the above thematic analysis information, but it is not yet clear why the Mimic game was so favored. One potential explanation is the feeling of accomplishment that users gained after mastering this more cognitively focused game, especially after failing to succeed in the initial attempt (as was the case for 18 participants). Among other activities, this game may have been more mentally engaging. We encourage robot-mediated exergame designers to consider a similar blending of physical and cognitive activities as one potential strategy for promoting game success (even in cases lacking in nostalgia or social references).

### 5.5 Key strengths

The results presented in this article move beyond our game-specific findings in Fitter et al. (2020) and toward a way to think about exercise games that may be more generalizable. Interested researchers could potentially build from our proposals about why the four most popular games were favored as such, using these ideas as hypotheses for future empirical work. We believe that the results indicated herein—that musical cultural references and socially engaging premises can supercharge robot-mediated exercise, and that these factors may even outweigh the clarity of gameplay—are crucial and merit careful consideration by both the rehabilitation robotics and the socially assistive robotics communities. Participants were typically successful in completing the exercise games, appearing to feel accomplishment particularly after winning a challenging game. Other researchers with a Baxter robot can apply this work directly by leveraging our open-source game repository (Fitter, 2020); specific lists of suggestions for further game iteration appear in Supplementary Appendix SB. The proposed games (especially with the recommended modifications) have the potential to positively impact human health by encouraging exercise.

### 5.6 Limitations

The study design was not without limitations. For example, the Strength game was so popular that opinions on this activity sometimes overwhelmed the responses to the other games. While this enthusiasm is a positive sign for using cultural references to build engagement, this effect could make examining what aspects of other games were engaging more difficult. The presence of a research team member during the study helped participants navigate games, but it also resulted in a potential increase in user confidence and comprehension during the games. In the future, it would be ideal to allow users to learn and play games independently (e.g., through instructions delivered via a tablet near the robot or through feedback provided by the robot itself) to gain a better understanding of how a system may be used outside of a lab setting, e.g., Mohan et al. (2021). This change to more independent gameplay, likely in less-structured environments and without closely linked compensation, could result in a lower chance of demand characteristics in the study design. There are also potential benefits of using wearable sensors to record user arm movement and physiological signals for objective measures of exercise, smart difficulty-level adaptation, or biofeedback. The reported level of exercise of participants was relatively low, despite the goals of the work. In the current work, we prioritized allowing users to be successful in their gameplay, which may have led to these lower exercise levels. In the future, we anticipate that we could simply increase game difficulty to yield greater exercise. Although the user population was diverse, we mostly lacked participants in the middle-age range, and we did not collect specific enough demographics to replicate (for example) the distribution of participant socio-economic status. The short-term and in-lab nature of the study also limit our understanding of the studied exergames; longer-term study in the wild and with more diverse users is needed to better investigate how the games perform and whether they see use past the point of novelty.

### 5.7 Conclusion

Overall, the results of this work show the promise of recognizable music and immersive, socially familiar experiences for robot-mediated exergames, in addition to hinting that engaging interactions require at least a moderate level of gameplay clarity. Importantly, cultural and social touchstones can be designed independently of the game mechanics and overlaid on a wide variety of games. The reasonably popular Mimic game further demonstrates that challenging activities that are nevertheless achievable for users may hold merit for exergame design. Although these ideas are already established for game design in general, they are not yet well-understood within the rehabilitation robotics space. Accordingly, there is a need to confirm that ideas of this type will replicate between application domains, and rehabilitation robotics researchers must assess whether and how existing game principles transfer to exergames. Researchers working on related topics should consider what our work suggests about the benefits of musical cultural touchstones, social experience, and immersion in the robot-mediated exercise game space. Although recent work has made strides in this area, the historical tendency in most rehabilitation robotics work has been to focus more on the physical mechanisms and control systems, and less on the social user experience. Our work indicates that a mix of reasonably clear system usability and some added social dimension is best to engage users, even in cases when one type of exercise is most important to the patient’s medical needs (e.g., the physical needs of someone with stroke or the cognitive needs of a user with dementia). This insight agrees with past assistive robotics work (e.g., Fasola and Matarić, 2012; Kashi and Levy-Tzedek, 2018) and signals a need for careful consideration of such a blend in rehabilitation robotics more broadly.

## Data Availability

The datasets presented in this article are not readily available because the video and text data that is the focus of the current article cannot be shared while ensuring anonymization of participant identity. However, most materials associated with this work are available for easy replication. A video explaining each exercise game is available here: https://www.youtube.com/watch?v=5zlaqlJJpts&feature=youtu.be. Individuals who are interested can download the source code for our exercise games here: https://github.com/shareresearchteam/baxter-exercise-games. In this repository, we include details on how to launch the exercise games in the same way that we did in our study. Requests to access the datasets should be directed to NF, naomi.fitter@oregonstate.edu.
