# Supplementary material for: How should robots exercise with people? Robot-mediated exergames win with music, social analogues, and gameplay clarity
Source: Front Robot AI. 2024 Jan 12;10:1155837. doi: 10.3389/frobt.2023.1155837 (PMC10813396; doi:10.3389/frobt.2023.1155837)
Supplement: Supplementary file 1 [file DataSheet1.pdf]

Appendices

A Video Annotation Codebook

This section details the codebook used in the video annotations presented in this article.

Codes Pertaining to All Games

- Number of Tries: number of times that the participant attempted a game
- Participant Verbal: spoken words by the participant
  - Task Clarification: requested clarifications about the game to help the participant gain a better understanding of the task at hand
  - Comment to Baxter: comment made directly to the robot
  - Comment on Robot: comment made to the experimenter about Baxter or its behavior
  - Comment on Game: verbal comment or feedback about any of the games or game designs/gameplay

Strength Game Codes

- Game Ending: the displayed Baxter facial expression and body language at the end of the game. The four performance tiers were joyful (ecstatic face, Baxter's arms in their highest position), happy (happy face, Baxter's arms up but lower than in joyful), neutral (neutral face, Baxter's arms roughly parallel to floor), and sad (afraid face, Baxter's arms sag toward floor)

Agility Game Codes

- Failed to Wake: participant failed to successfully complete the game and wake Baxter
- Continued Hitting: participant continued to hit even after Baxter awoke

Mimic Game Codes

- Human Error: incorrect movement performed by the participant
- Robot Error: robot detection of a movement that did not actually happen (false positive) or failure to register a correct participant movement (false negative)
- Human Win: scenario where participant won the game
- Robot Win: scenario where robot won the game

Roboga Game Codes

- Did Not Relax: participant did not relax during the neutral/relaxed pose included periodically in gameplay
- Mistake: participant made an error in imitating the hand position or orientation of Baxter's pose

Handclap Game Codes

- Incorrect Clap: participant performed a forward clap instead of a cross-body clap or vice versa
- Missed Clap: participant did not hit when they should have
- Human Win: participant successfully reached the end of the game
- Human Loss: participant did not reach the end of the game

Teach Game Codes

- Number of Chords: count of the total chords explored by the participant

Stretch Game Codes

- Robot Error: even though the participant hit, the robot registered a miss (false negative)

Flamenco Game Codes

- Extra Move: participant performed an extra move not included in Baxter's choreography
- Missed Move: participant skipped a move while reproducing Baxter's dance sequence
- Wrong Move: participant performed an incorrect move in the dance sequence

B Future Game Improvements

Participants had many explicit suggestions and constructive criticisms for the games, some of which align directly with user comments mentioned in the body of this article. We view this feedback as part of a larger human-centered design process surrounding the work; we have engaged in several design cycles during the project so far, and we imagine that future users of our open-source exercise game repository (further described in [?]) will likewise follow this process. A summary of key feedback for future design cycles of each game appears below.

- Strength Game: louder cues, ability to choose song, rewards for harder hits, more hand poses, asymmetric hand poses, more introductory information about possible performance levels
- Agility Game: earlier indicator of feedback, omission of red face color, more continuous face color scale, Baxter hand position movement during play
- Mimic Game: more actions, faster actions, different rhythms, timing help from a metronome, clearer game end cues, longer play
- Roboga Game: asymmetric poses, rhythmic motions, yoga music, omission of red face color, active feedback about performance, longer or shorter held poses to vary exercise intensity
- Handclap Game: pause between teaching and playing, auditory feedback when transitioning between phases, removing the teaching phase
- Stretch Game: recognizable song, faster actions, harder-to-reach poses, happier-sounding music, cat-and-mouse style game mode in which the robot tries to evade the human's touch, ability to choose song

- Flamenco Game: visual indicator of next move, faster moves, slower moves, clearer start and end cues, physical interaction with the robot, active feedback about performance, more recognizable music
- Teach Game: visual scale indicator on Baxter's face, more help picking nice chords or writing a recognizable song, ability to record single notes, way to test chords, way to go back and replay and/or delete notes

As our aim in the current study was to set game challenge at a level attainable for most participants, it seems that we may have aimed too low with the default difficulty of certain games. From the default level used for each game in the study, difficulty can be adjusted to be more or less challenging (for example, via a need for faster input actions, more impact force, longer patterns to memorize, longer held poses, or lengthier compositions). Based on the user feedback, we encourage parties who are interested in our open-source games to consider the settings of the current Agility, Handclap, Mimic, Roboga, and Strength games as reasonable starting levels, but researchers should immediately increase the challenge of the Flamenco, Stretch, and Teach games in their work.
